# Colovesical Fistula in a Kidney Transplant Patient: A Fatal Outcome

**DOI:** 10.7759/cureus.71145

**Published:** 2024-10-09

**Authors:** Guilherme C Gonzales, Rafael S Aguiar, Vincius C Lopes, Luisa F De Arruda, Ricardo H de Rizzo, Henrique R Cortines, Gabriel A Agareno, Andre L Coelho Pereira, Pedro Francisco F De Arruda

**Affiliations:** 1 Urology, Faculty of Medicine of São José do Rio Preto, São José do Rio Preto, BRA; 2 Medicine, Ribeirão Preto Medical School, Ribeirão Preto, BRA

**Keywords:** colovesical fistula, emphysematous pyelonephritis, immunosuppression, kidney transplantation, renal abscess, urinary infection

## Abstract

Colovesical fistula (CVF) is a rare but potentially serious condition characterized by abnormal communication between the colon and the bladder. This pathology can result from inflammatory diseases, malignancies, or previous surgical interventions, with a significant impact on the patient's quality of life. CVF is associated with high morbidity and mortality rates, particularly in immunosuppressed individuals, such as renal transplant recipients, due to their increased susceptibility to infections and surgical complications. In this case report, we describe a case of a 57-year-old male patient, a renal transplant recipient under chronic immunosuppressive therapy, who developed an enterovesical fistula. The management of CVF in transplant patients poses diagnostic and therapeutic challenges, requiring a multidisciplinary approach to optimize clinical outcomes. The relevance of this case lies in the complexity of treating a severely ill immunosuppressed patient, with few similar cases reported in the literature.

## Introduction

Colovesical fistula in patients having been submitted to kidney transplantation is a rare condition with few reported cases and is associated with high rates of morbidity and mortality. The most common cause of this condition is diverticulitis of the sigmoid colon, followed by Crohn’s disease, malignant neoplasms, radiotherapy, and trauma [1].

Although rare, this condition is potentially fatal, especially in transplanted patients, requiring an early diagnosis and adequate therapeutic approach. This report describes a case of colovesical fistula in a kidney transplant recipient involving renal abscesses, the need for urgent surgery, septic shock, and death.

## Case presentation

A 57-year-old male patient having been submitted to kidney transplantation (cadaver donor) 11 months earlier with a history of pulmonary tuberculosis sought medical care due to abdominal pain with a one-month history and recent worsening associated with nausea and hyporexia. The physical examination revealed diffuse abdominal pain upon palpation but with no signs of peritoneal irritation.

Laboratory tests at admission revealed a significant increase in inflammatory tests and signs of sepsis. The results are described in Table 1.

**Table 1:** Results of laboratory tests performed on admission.

Blood and urine tests	Admission	Reference values
Hemoglobin	10.9 g/dL	12.8 to 16.5 g/dL
Leukocytes	18200/mm3	4000 to 11000/mm3
Platelets	69000/mm3	140000 to 450000/mm3
Sodium	145 mmol/L	135 to 145 mmol/L
Potassium	4.50 mmol/L	3.50 to 5.10 mmol/L
Urea	79 mg/dL	Below 50 mg/dL
Creatinine	2.34 mg/dL	0.60 to 1.20 mg/dL
C-reactive protein	30.99 mg/dL	Below 0.50 mg/dL
Procalcitonin	23.19 ng/mL	Below 0.50 ng/mL: low risk of sepsis and/or septic shock; higher than 2.00 ng/mL: high risk of sepsis and/or septic shock
Urine test	Density: 1010	1015-1025
	pH: 6.0	5.0-7.0
	Nitrites: Negative	Negative
	Leukocytes: 11000 /mL	Up to 25000/ mL
	Erythrocytes: 7000 /mL	Up to 25000/ mL
	Bacteria: Rare	Rare
	Crystals: Absent	Absent
Urine culture	Negative	

**Figure 1:**
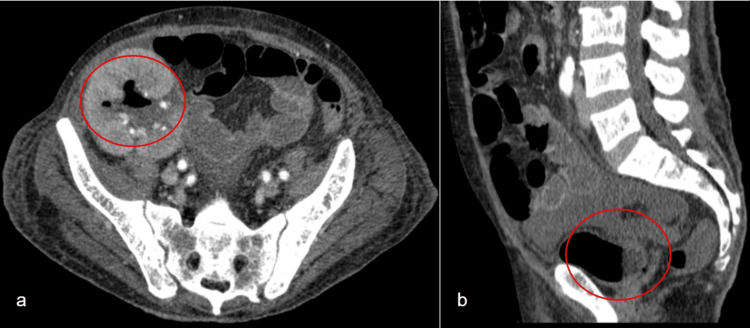
Tomogram showing the presence of gas in transplanted kidney and bladder. Figure (a) shows an axial scan highlighting the presence of gas in the transplanted kidney. Figure (b) shows a sagittal scan highlighting the presence of gas in the urinary bladder.

**Figure 2:**
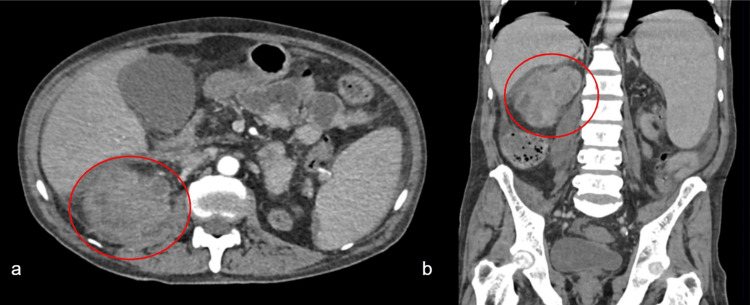
Tomogram demonstrating pyelonephritis and renal abscesses in the right primitive kidney. Figure (a) shows an axial scan and figure (b) shows a coronal scan. In both figures, pyelonephritis and renal abscesses in the right primitive kidney are highlighted.

Computed tomography of the abdomen revealed a transplanted kidney located in the right iliac fossa with increased dimensions and edematous parenchyma representing pyelonephritis; globous right primitive kidney with renal abscesses of up to 4.3 cm and gaseous pockets in the interior of the collector system (Figures 1, 2), representing probable emphysematous pyelonephritis, with an appearance of emphysematous cystitis; discrete, reactional colitis to the right near the transplanted kidney observed in the proximal third of the ascending colon, with parietal thickening and blurring of the contours.

The patient was admitted by the nephrology team and treatment for emphysematous pyelonephritis was initiated. However, the patient exhibited important clinical worsening and nephrectomy was performed in the right primitive kidney due to the presence of an abscess.

**Figure 3:**
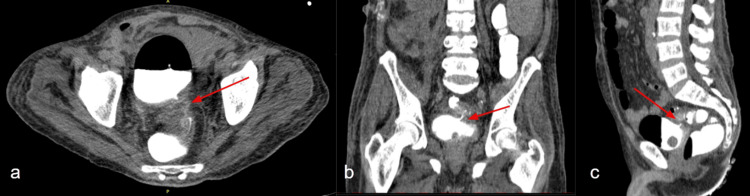
Tomogram showing colovesical fistula and present of enteral contrast. Figure (a) shows an axial scan, figure (b) shows a coronal scan, and figure (c) shows a sagittal scan. In all of them, the fistulous path is highlighted.

The patient remained in intensive care, with high inflammatory tests and the growth of *Klebsiella pneumoniae* in the urine culture sensitive only to gentamycin and ceftazidime/avibactam. A new tomographic study was performed of the abdomen, which revealed an abscess at the surgical site and the presence of gas in the bladder as well as a colovesical fistula (Figure 3) following the intravesical injection of contrast.

Despite transversostomy performed by the proctology team and culture-guided antibiotic therapy, the patient did not exhibit significant clinical improvement and died about 30 days after admission due to refractory septic shock.

## Discussion

Although rare, colovesical fistula accounts for approximately 80% of uro-digestive fistulas, the most common of which is a fistula between the bladder and sigmoid colon. Diverticulitis is the most frequent cause of colovesical fistula (50-70% of cases), followed by Crohn’s disease and malignant colorectal neoplasms [1]. The proportion between men and women is 3:1. The lower incidence among women is due to the fact that the uterus is situated between the bladder and sigmoid colon [2].

The clinical condition mainly involves pneumaturia, fecaluria, and recurrent urinary infections. The occurrence of pneumaturia is a highly specific sign of communication between the intestine and bladder and is reported in 60-85% of cases described in the literature [3]. In the case described here, the patient had none of these complaints at the time of assessment.

The diagnosis can be challenging and involves different exams, such as contrast exams (e.g., urethrocystography, barium enema, and computed tomography of the abdomen with contrast), ultrasound, and even endoscopic exams, such as cystoscopy [4]. The diagnosis in the present case was made by cysto-tomography.

Fistula can be treated in different ways. In select cases, conservative treatment can be considered, which involves indwelling bladder catheterization, a parenteral diet, and other aspects. However, most patients require surgical correction [4,5].

In the case described here, a nephrectomy of the primitive kidney was initially performed due to the presence of an abscess. However, after the definitive diagnosis of the fistula, the treatment option was a deviation of the intestinal transit by transversostomy due to the patient’s clinical condition, which impeded more aggressive interventions. Ideally, the affected intestinal segment should be resected at the same surgical time, if possible [6,7].

The literature offers few reports of colovesical fistula in kidney transplant recipients [8-10]. It should be pointed out that diverticulitis tends to be more frequent in patients undergoing dialysis compared to the general population [8]. Although the clinical condition and diagnosis are similar to those in non-immunosuppressed patients, the clinical outcome can be fatal, as occurred in the case described here. Furthermore, such patients may have multi-resistant microbiota due to recurrent urinary infections, hindering an effective response in cases of urinary sepsis.

## Conclusions

Early diagnosis of colovesical fistula is crucial, particularly in immunosuppressed patients, such as renal transplant recipients. Prompt recognition of this condition can prevent severe complications and optimize clinical outcomes, reducing the risk of recurrent and multidrug-resistant infections, which, as observed in the described case, are associated with high mortality. The severity of the clinical presentation and the high susceptibility to infectious complications make it imperative to consider colovesical fistula as a differential diagnosis in immunosuppressed patients presenting with suggestive symptoms, such as recurrent urinary tract infections, pneumaturia, and fecaluria. Additionally, due to the rarity of reported cases of colovesical fistula in renal transplant patients in the literature, this case underscores the need for increased awareness and diagnostic vigilance to ensure effective treatment and reduce morbidity and mortality. The prognosis directly depends on how quickly the diagnosis is made and the adequacy of the treatment, reinforcing the importance of including this condition in the differential diagnosis of patients with similar risk factors.
